# Adenine base editor corrected ADPKD point mutations in hiPSCs and kidney organoids

**DOI:** 10.1007/s44307-024-00026-8

**Published:** 2024-06-11

**Authors:** Jingwen Wang, Yanling Qiu, Lei Zhang, Xinyao Zhou, Sihui Hu, Qianyi Liu, Sisi Yin, Zehong Su, Simiao Liu, Haiying Liu, Xueqing Wu, Junjiu Huang

**Affiliations:** 1 MOE Key Laboratory of Gene Function and Regulation, State Key Laboratory of Biocontrol, School of Life Sciences, Sun Yat-Sen University, Guangzhou, Guangdong 510275 China; 2 Center of Reproductive Medicine, Children’s Hospital of Shanxi and Women Health Center of Shanxi, Taiyuan, Shanxi 030013 China; 3 Key Laboratory of Reproductive Medicine of Guangdong Province, The First Affiliated Hospital and School of Life Sciences, Sun Yat-Sen University, Guangzhou, Guangdong 510275 China

**Keywords:** Gene editing, ABE, hiPSCs, Kidney organoid, ADPKD

## Abstract

Autosomal dominant polycystic kidney disease (ADPKD) is a dominant genetic disorder caused primarily by mutations in the *PKD1* gene, resulting in the formation of numerous cysts and eventually kidney failure. However, there are currently no gene therapy studies aimed at correcting *PKD1* gene mutations. In this study, we identified two mutation sites associated with ADPKD, c.1198 (C > T) and c.8311 (G > A), which could potentially be corrected by adenine base editor (ABE). The correction efficiencies of different ABE variants were tested using the HEK293T-*PKD1* c.1198 (C > T) and HEK293T-*PKD1* c.8311 (G > A) reporter cell lines. We then generated induced pluripotent stem cells (iPSCs^mut/WT^) from the peripheral blood mononuclear cells (PBMCs) of the heterozygous patient to develop a disease cell model. Since the iPSCs^mut/WT^ did not exhibit a typical disease phenotype in stem cell status, differentiation into kidney organoids in vitro led to the expression of kidney organ-specific marker proteins. Stimulation of cAMP signaling with forskolin resulted in cystic expansion of renal epithelial tissue in iPSC^mut/WT^-derived kidney organoids, resembling the cystic phenotype observed in ADPKD patients. However, kidney organoids differentiated from ABE-corrected iPSCs did not display the cystic phenotype. Furthermore, we used a dual AAV split-ABEmax system as a therapeutic strategy and achieved an average editing efficiency of approximately 6.56% in kidney organoids. Overall, this study provides a framework for gene therapy targeting ADPKD through ABE single-base editing, offering promising prospects for future therapeutic interventions.

**Supplementary Information:**

The online version contains supplementary material available at 10.1007/s44307-024-00026-8.

## Introduction

Autosomal dominant polycystic kidney disease (ADPKD) is the most common and potentially fatal monogenic disorder and the most common inherited kidney disease (Torres et al. 2007). The prevalence of ADPKD is estimated to be 1/1000–1/2500 (Lanktree et al. 2018; Willey et al. 2017), and occurs in all regions of the world and in all ethnic groups, but the incidence rate varies by region. In the United States, about 5% of dialysis patients each year have kidney disease caused by ADPKD (Cornec-Le Gall et al. 2019; Harris and Torres 2009). Approximately 1.5 million people in China suffer from the disease (Xue et al. 2016). ADPKD has several extrarenal manifestations, including hepatic cysts, pancreatic cysts, intracranial aneurysms, colonic diverticulosis and cardiovascular defects, which can be systemically life-threatening (Xue et al. 2016). ADPKD tends to manifest more severely in males compared to females (Conte et al. 2023; Shukoor et al. 2021; Xue et al. 2016). Unfortunately, there are currently no effective treatments for ADPKD.

Two genes have been identified that are associated with ADPKD, *PKD1* (approximately 85% of cases) and *PKD2* (approximately 15% of cases) (Torres et al. 2007). *PKD1*, located on chromosome 16, encodes polycystin-1 (PC1), a transmembrane binding protein of 4303 amino acids with 11 transmembrane domains, a large extracellular N-terminal domain and a short (about 200 amino acids) cytoplasmic C-terminal domain (Consortium 1995; Hughes et al. 1995). The C-terminal domain of PC1 interacts with polycystin-2 (PC2), a 968 amino acid protein encoded by *PKD2*, which is a member of the transient receptor potential (TRP) channel family (Hayashi et al. 1997). Somatic “second hit” mutations targeting the wild-type allele of either gene in kidney tubule cells have been shown to initiate cysts, followed by the involvement of additional factors that regulate the cyst progression (Dong et al. 2021; Leonhard et al. 2016; Wu et al. 1998). After knockout of *Pkd1* or *Pkd2* in mice, homozygous genotypes showed cardiovascular defects and embryonic lethality, whereas heterozygous genotypes developed progressive renal and hepatic cysts, consistent with what has been observed in humans (Boulter et al. 2001; Kim et al. 2000; Lantinga-van Leeuwen et al. 2007; Lu et al. 1999, 2001). PC1-PC2 complexes can be detected in the endoplasmic reticulum membrane, plasma membrane, exosomes and primary cilia. It is generally believed that cilia are at the core of ADPKD pathogenesis, but the function of the PC1-PC2 complex and its role in the pathogenesis of ADPKD is unclear.

With the development of human induced pluripotent stem cells (hiPSCs) and organoid technologies, more and more studies have used disease-specific hiPSC-induced kidney organoids to elucidate the mechanism of early disease onset in ADPKD. In 2020, Nishida et al. used CRISPR/Cas9 gene editing technology to edit the 34th exon of *PKD1* gene, and obtained two hiPSC cell lines with *PKD1* homozygous and heterozygous mutations (Nishida et al. 2016). It was observed that forskolin could induce vesicles produced in the *PKD1* mutant group after 7 days, and the area and number of vesicles in the homozygous mutant group far exceeded that of the heterozygous mutant. Another study extended the disease model of ADPKD by using a *PKD1*-deficient hiPSC line to differentiate ureteral bud organoids and develop cysts in response to cAMP signaling (Facioli et al. 2021). However, the aforementioned are all cell lines in 2D, and a systematic model is currently unavailable.

To date, there have been no reports of gene therapy for ADPKD. According to the updated ADPKD Variant Database (http://pkdb.mayo.edu/), a large proportion of ADPKD *PKD1* mutations have been found to be single base mutations, therefore CRISPR-Cas9-based single-nucleotide editing technologies may offer the opportunity to correct these precisely. Previous research has shown that the incidence of off-target effects is significantly lower with adenine base editors (ABEs) compared to cytosine base editors (CBEs) or CRISPR/Cas9 (Liang et al. 2019; Zuo et al. 2019). Recently, we found that the ABE can correct CADASIL point mutations in hiPSCs and blood vessel organoids without significant off-target effects (Wang et al. 2024). This suggests that ABEs may be a safer option for clinical therapeutic applications. Based on previous research results in our laboratory, we chose ABE7.10 and ABEmax, which have relatively high editing efficiencies and low off-target effects (Wang et al. 2024).

This study attempted to use an ABE to repair two clinically discovered pathogenic *PKD1* point mutations. We found that there was no editing effect on the *PKD1* (c.1198 C > T) point mutation, while the *PKD1* (c.8311 G > A) site could be efficiently repaired by ABEmax. After hiPSCs were differentiated into kidney organoids in vitro, the mutant group produced multiple vesicles upon forskolin stimulation, reduced the number of mitochondria in kidney tissue cells and showed abnormal proliferation of kidney epithelial cells. Using the dual adeno-associated viruses (AAV)-mediated Split-ABE system to infect kidney organoids, the average editing efficiency reached 6.56 ± 0.52% at 20 days, and the highest editing efficiency reached 9.48%. In conclusion, we established an iPSC-based ADPKD model and corrected *PKD1* mutations using a dual AAV Split-ABEmax system. It was demonstrated for the first time that ABE can directly repair ADPKD point mutations in kidney tissue, providing a research basis for the clinical treatment of this disease.

## Materials and methods

### Cell lines and cell culture

HEK293T cells were cultured in DMEM medium (Corning) supplemented with 10% fetal bovine serum (FBS) (Excell, Australia). Human induced pluripotent stem cells (hiPSCs) were generated by reprogramming peripheral blood mononuclear cells (PBMCs) using of the CytoTune-iPS 2.0 Sendai Reprogramming Kit (Thermo, A16517). H1 hESCs and hiPSCs were cultured in mTeSR1 medium (STEMCELL, 85850).

### Generation of stable cell lines

Two DNA fragments containing the c.1198 (C > T) and c.8311 (G > A) mutations within the *PKD1* sequence were cloned individually into the pENTR vector. These fragments were then ligated into the pLenti vector using the Gateway^®^ LR clonase reaction (Thermo Fisher, 11791020). After lentiviral transduction of wild-type HEK293T cells for 72 h, puromycin was used for drug selection. Single clones with either c.1198 (C > T) or c.8311 (G > A) mutations were then isolated and subjected to PCR-based detection using primers listed in Table S1. A cell line integrating the specific fragment (c.1198 (C > T) or c.8311 (G > A) mutations) was selected for subsequent experimentations.

### Reprogramming PBMCs to hiPSCs

Peripheral blood mononuclear cells (PBMCs) were isolated from the patient’s blood by density gradient centrifugation. These cells were cultured in StemProTM-34 medium (Gibco, 10639-011) supplemented with 2 mM L-glutamine (Life technologies, 35050038), 100 ng/mL SCF (Gibco, PHC2111), 100 ng/mL FLT-3 (Gibco, PHC9414), 20 ng/mL IL-3 (Gibco, PHC0034) and 20 ng/mL IL-6 (Gibco, PHC0065). The cells were then transduced with the CytoTune-iPS 2.0 Sendai reprogramming vectors in CytoTune™-iPS 2.0 Sendai Reprogramming Kit (Thermo, A16517) and then seeded onto a feeder layer of mouse embryonic fibroblasts (MEFs). The emergence of hiPSC colonies was observed within approximately 12 days. Once the hiPSC colonies were ready for transfer, undifferentiated hiPSCs were manually picked and transferred to Matrigel-coated (BD, 356230) culture dishes to facilitate their expansion. The hiPSCs were maintained in a feeder-free environment, using Matrigel-coated culture dishes and cultured in mTeSR1 medium. Routine passaging was performed using 0.5 mM EDTA diluted in DPBS (Gibco, 14190144) without Ca^2+^/Mg^2+^. For single cell passaging, 10 μM Y27632 (Santa Cruz, sc-281642A) was added.

### Ethics

Human blood samples were obtained from the Center of Reproductive Medicine, Children’s Hospital of Shanxi and Women Health Center of Shanxi with written consent from the donors. All procedures adhered to the ethical standards of the Ethic Committee of Children’s Hospital of Shanxi and Women Health Center of Shanxi (Approval Reference Number: IRB-KYHZ-2019-008) and with the Helsinki Declaration of 1975, as revised in 2000.

### Genome editing using the ABE system

Each ABE variant was individually cloned into a mammalian expression vector containing a CMV promoter. These constructs were then co-transfected with the pUC19-gRNA plasmid into HEK293T cells using polyethyleneimine (PEI). In each well of a 6-well plate, 3 μg of plasmid DNA was used, with a 2:1 ratio of ABE to gRNA. For hiPSCs, the same quantity of plasmid DNA was used for electroporation utilizing the P3 Primary Cell 4D-Nucleofector Kit (Lonza, V4XP-3032). After transfection, the hiPSCs were seeded onto Matrigel-coated 12-well plates and cultured in mTeSR1 medium supplemented with 10 μM Y2763 for 72 h before detection. Single hiPSCs were then cultured in mTeSRplus medium (STEMCELL, 05825) supplemented with CloneR (STEMCELL, 05889). After approximately 10 days, single clones were selected and expanded for genotyping by Sanger sequencing.

### Off-target analysis

The top ten potential off-target sites were identified using Cas-Offinder and PCR amplification was performed for subsequent deep sequencing. The PCR products were subjected to paired-end 150 sequencing using the NovaSeq 6000 platform (Illumina) at Annoroad Gene Technology (Beijing, PR China) Co., Ltd. The single-nucleotide transversion editing efficiency was quantified and analyzed using MATLAB.

### Quantitative real-time PCR (RT-qPCR)

Total RNA was extracted using the phenol-chloroform method. Subsequent cDNA synthesis was performed using the Prime-ScriptTM RT Reagent Kit (Takara, RR047A). For quantitative real-time PCR (RT-qPCR), TB Green Premix EXII (Tli RnaseH Plus) (Takara, RR820A) was utilized together with qPCR primers. The qPCR was performed on an ABI StepOnePlus real-time PCR system. The list of primers used can be found in Table S1.

### Immunofluorescence

Cells were plated on 15 mm microscope coverslips and fixed with 4% paraformaldehyde (PFA, 158127) at ice-cold temperatures for 30 min. The cells were then permeabilized (using a solution containing 5% Triton-X, 20 mM HEPES, 3 mM MgCl_2_, and 300 mM sucrose in PBS) for 30 min at 25℃. After permeabilization, a blocking step was performed for 1 h at RT (using a solution containing 3% goat serum and 0.1% BSA in PBS), followed by an overnight incubation with primary antibodies at 4 °C. After primary antibody incubation, cells were stained with Alexa Fluor-conjugated secondary antibodies (Alexa Fluor 488 goat anti-mouse IgG (Invitrogen, A-11017, 1:500) or Alexa Fluor 555 donkey anti-rabbit IgG (Invitrogen, A-31570, 1:500)), for 1 h at RT. Nuclear staining was performed with DAPI. Antibodies used for immunofluorescence included anti-OCT4 (mouse, BD 611202), anti-NANOG (rabbit, Santa Cruz D1021), anti-SOX2 (mouse, BD 561469), anti-SSEA4 (mouse, Invitrogen RL239281), anti-Ki67 (rabbit, ab16667), CDH1 (mouse, BD610182), LTL (Vector Laboratories, FL1321).

### hiPSCs differentiate into three germ layers in vitro

To induce definitive endoderm differentiation, hiPSCs were plated in mTeSR1 medium supplemented with 5 ng/mL Recombinant Human BMP-4 (hBMP4, PeproTech 120-05ET), 25 ng/mL activin A (PeproTech, 120-05ET), and 1 μM CHIR99021 (Cayman, 13122) for a period of 2 days. CHIR99021 was then removed for the remainder of the differentiation process. Cells were harvested for analysis after 7 days of differentiation. To induce ectoderm differentiation, hiPSCs were cultured in MEF conditioned medium supplemented with 0.1 mM LDN193189 (Selleck, S2618) and 10 mmol/mL SB431542 (Sigma, 616464). Cells were collected after 7 days of culture. To induce mesoderm differentiation, hiPSCs were cultured in N2B27 medium supplemented with 8 μM CHIR99021 and 25 ng/mL hBMP4. Cells were collected after 3 days of culture.

### Kidney organoid differentiation from hESC/hiPSC

Accutase was used to dissociate H1 hESC/hiPSC into single cells, which were placed in a 12-well plate treated with Matrigel at an inoculation density of 15,000 to 24,000 cells/cm^2^ and cultured for 3–4 days until the cell density was 50%-60%., referred to as Day 0. The differentiation line was then induced. The medium was replaced with the basal differentiation medium (RPMI 1640 medium with 10% KSR (Gibco, 10828-028), 2 mM L-GlutaMAX (Gibco, 35050061), 0.1 mM minimum essential medium (MEM) non-essential amino acids (Gibco, 11140050)) and add 10 μM CHIR99021 and cultured for 4 days with daily medium changes. Induction of renal-derived mesodermal cell differentiation. The culture medium was changed to a factor-free basal differentiation medium and cultured for 3 days with daily medium changes. Induction of nephron progenitor cell differentiation. The culture medium was changed to basal differentiation medium, 50 ng/mL FGF9 (PeproTech, 100-23) and 3 mM CHIR99021 were added, cultured for 2 days with daily medium changes. On day 10 of differentiation, the medium was changed to basal differentiation medium and 50 ng/mL FGF9 was added. On day 14 of differentiation, the culture medium was changed to basal differentiation medium and 50 ng/mL FGF9 and 1 mM CHIR99021 were added to adjust the ratio of the proximal to distal segments. From day 20 of differentiation, the basal differentiation medium was used for organoid culture. To generate 3D kidney organoids, cells were digested into single cells by accutase (STEMCELL, 7920) on day 10–12. 2.5–5 × 10^4^ cells were inoculated into a 96-well plate with a circular bottom and low adhesion. On day 16–18 of differentiation (5–7 days after aggregation), 3D kidney organoids were transferred to the upper chamber of the transwell (Corning, 3450) for liquid-air interface culture and the culture medium was changed daily in the lower chamber.

### Cell cycle analysis

Cells were harvested at 60% confluence and fixed in 75% ethanol at -20 °C overnight. Cells were washed with PBS and resuspended in 200 μL PBS. After the addition of 0.2 mg/mL RNase A (Takara, 2158), the cells were digested at 37 °C for 30 min. Flow cytometry analysis was then performed using the BECKMAN CytoFLEX system and cell-cycle phases distributions were evaluated using ModFitLT software.

### Flow cytometry analysis and cell sorting (FACS)

Cells were harvested, washed once with PBS and resuspended in 200 μL of serum-free medium. FACS sorting was performed using FACSAria or FACSAriaIII (BD) cell sorter and results were analyzed using FACSDiva (BD) or Flowjo (Tree Star) software.

### Statistical analysis

Two-way analysis of variance (ANOVA) was performed to compare the mean of more than two groups. Unpaired Student’s t test was used to analyze the mean of the two groups. Error bars represented the standard error of the mean (SD) of three independent experiments, and significant differences were defined as *, *p* < 0.05, **, *p* < 0.01, and ***, *p* < 0.001.

## Results

### Screening of optimal variant ABEs with *PKD1* c.1198 (C > T) and c.8311 (G > A) mutation reporter cell lines

**Fig. 1:**
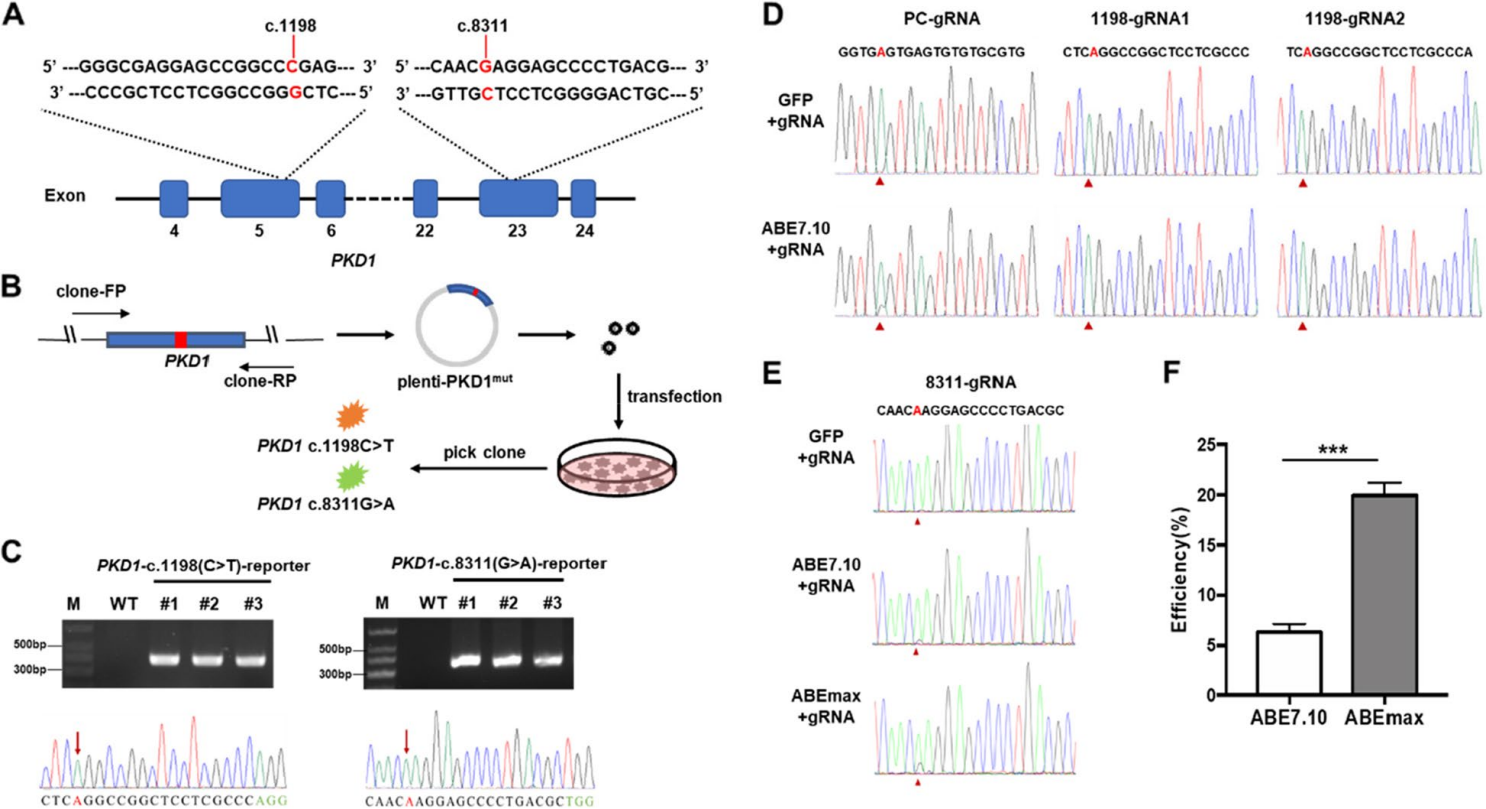
Generation of 293T-*PKD1* c.1198 (C > T) and c.8311 (G > A) reporter cell lines and detection of the editing efficiency of ABE variants. **A** Schematic representation of the *PKD1* c.1198 (C > T) and c.8311(G > A) mutations. **B** Workflow of generation of 293T-*PKD1* c.1198 (C > T) and c.8311 (G > A) reporter cell lines. **C** Identification of 293T-*PKD1* c.1198 (C > T) and c.8311 (G > A) reporter cell lines by PCR amplification and Sanger sequencing. **D** Sanger sequencing results of the ABE7.10 editing in the 293T-*PKD1* c.1198 (C > T) reporter cell line, the mutated adenine is marked with a red arrow. **E** Sanger sequencing results of ABE7.10 editing in 293T-*PKD1* c.8311 (G > A) reporter cell line. **F** Statistical results of editing efficiency of ABE7.10 and ABEmax in 293T-*PKD1* c.8311 (G > A) reporter cell line. Error bars indicate SD (*n* = 3). Statistical significance was determined by the two-tailed Student’s t test. *** *p* < 0.001

To determine whether the c.1198 (C > T) and c.8311 (G > A) mutations in *PKD1* could be corrected by using the ABE system, we first established HEK293T-*PKD1* c.1198 (C > T) and c.8311 (G > A) mutation reporter cell lines (Fig. 1A). The *PKD1* gene fragments with single mutations were packaged into lentivirus and two single clones were selected after puro selection (Fig. 1B). Polymerase chain reaction (PCR) using an external primer confirmed the insertion of *PKD1* segments, and Sanger sequencing also showed that the two loci were homozygous (Fig. 1C). Due to the limited editing window of the ABE system, we could only design two guide RNAs at the c.1198 (C > T) site and one guide RNA at the c.8311 (G > A) site (Fig. 1D, E). First, we co-transfected ABE7.10 and gRNA1, or ABE7.10 and gRNA2 into the HEK293T-*PKD1* c.1198 (C > T) reporter cell line, and collected DNA within 72 h. Sanger sequencing results showed that the c.1198 (C > T) site was not edited by gRNA1 or gRNA2 (Fig. 1D), indicating that the ABE single base editor cannot repair the *PKD1* c.1198 (C > T) point mutation. We then co-transfected ABE7.10 and gRNA into the HEK293T-*PKD1* c.8311 (G > A) reporter cell line. The results showed overlapping peaks at the c.8311 (G > A) site, proving that this mutation could be corrected by the ABE system (Fig. 1E). To improve the safety and editing efficiency of clinical applications, we tried to search for an ABE variant with the highest efficiency on this target site and no bystander editing effect. According to our previous research (Wang et al. 2024), the AEBmax variant is a promising candidate, and we tested it. The results indicate that AEBmax has a higher editing efficiency for the c.8311 site (Fig. 1E). The statistical results showed that the editing efficiency of ABE7.10 was only 6.34 ± 0.27%, while the editing efficiency of ABEmax was as high as 19.95 ± 0.44% (*** *P* < 0.001, Fig. 1F). Therefore, we used *PKD1* c.8311 (G > A) and ABEmax in the subsequent experiments.

### Derivation and correction of hiPSC lines from a *PKD1* c.8311(G > A) mutant patient

**Fig. 2:**
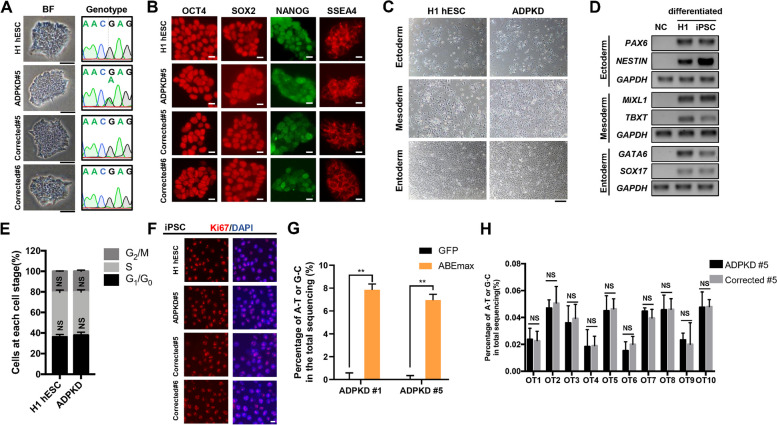
Establishment of hiPSCs and correction of the *PKD1* c.8311(G > A) mutation in hiPSCs. **A** The Sanger sequencing results confirmed the presence of the *PKD1* c.8311(G > A) mutation in ADPKD hiPSCs (left). In addition, the successful correction in hiPSC clones was demonstrated by Sanger sequencing (right). Bright field images of hiPSCs were shown on the left. Scale bar, 200 μm. **B** Immunofluorescence staining showed the pluripotency markers, including OCT4, SOX2, NANOG, and SSEA4. Scale bar, 100 μm. **C** Ectoderm, mesoderm, and endoderm differentiation of H1 hESCs and ADPKD hiPSCs were shown in bright field images. Scale bar, 200 μm. **D** RT-qPCR showed the expression of germ layer markers. *PAX6* and *NESTIN* for ectoderm, *GATA6* and *SOX17* for endoderm, and *MIXL1* and *TBXT* for mesoderm. Undifferentiated H1 hESCs/hiPSCs were used as negative controls. **E** Cell cycle analysis of H1 hESCs and ADPKD hiPSCs. Error bars indicate SD (*n* = 3). NS indicated not significant. **F** Immunofluorescence staining of Ki67 in ADPKD hiPSCs. Cell nuclei were stained with DAPI. Scale bar, 100 μm. **G** Statistical results of deep sequencing after electroporation at 72 h. Two ADPKD hiPSC clones (#1 and #5) were detected. Statistical significance was determined using the two-tailed Student’s T-test. **p < 0.01. **H** Statistical results of deep sequencing in predicted off-target sites. ADPKD clone #5 and Corrected clone #5 were detected. Error bars indicate SD (*n* = 3). NS indicated not significant

To establish an in vitro ADPKD model, peripheral blood mononuclear cells (PBMCs) were isolated from an ADPKD patient by density gradient centrifugation. These PBMCs were then reprogrammed into human induced pluripotent stem cells (hiPSCs) by infection with Sendai virus (SeV). The resulting hiPSC lines showed a typical morphology. The presence of compound heterozygous *PKD1* mutations in the hiPSCs was confirmed through Sanger sequencing (Fig. 2A). Immunofluorescence staining revealed high expression levels of OCT4, SOX2, NANOG, and SSEA4 in the induced ADPKD hiPSCs (Fig. 2B). The intensity of their expression was very similar to that of the positive control group, H1 hESC, indicating the robust pluripotency of ADPKD hiPSCs. We substantiated their pluripotent capability by effectively prompting differentiation into the three germ layers (Fig. 2C, D). In addition, cell cycle dynamics and Ki67 immunofluorescence staining revealed that the proliferative capability of the hiPSCs was similar to that of H1 hESCs (Fig. 2E, F). In summary, we have successfully generated ADPKD hiPSCs with normal pluripotency and robust proliferative capacity.

We further evaluated the editing efficiency of the ABE system in hiPSCs. After 72 h of electroporation, cells were harvested and editing efficiency was determined as the proportion of A:T to C:G conversion. Within two different ADPKD hiPSC single clones (ADPKD #1 and ADPKD #5), ABEmax showed correction rates of 7.84 ± 0.52% and 7.29 ± 0.51% respectively (Fig. 2G). To identify potential off-target effects, we selected two corrected clones (Corrected #5 and Corrected #6) from the ADPKD #5 group treated with ABEmax and gRNA (Fig. 2A, B and F). We used Cas OFFinder to predict off-target sites, and deep sequencing analysis showed that among the top 10 predicted sites, neither ADPKD #5 clone nor Corrected #5 clone had substantial off-target effects (Fig. 2H). Taken together, these results demonstrated the efficiency and safety of ABEmax for the correcting the *PKD1* c.8311(G > A) mutation in hiPSCs.

### Differentiation of mutant *PKD1* c.8311(G > A) hiPSCs into a kidney organoid

**Fig. 3:**
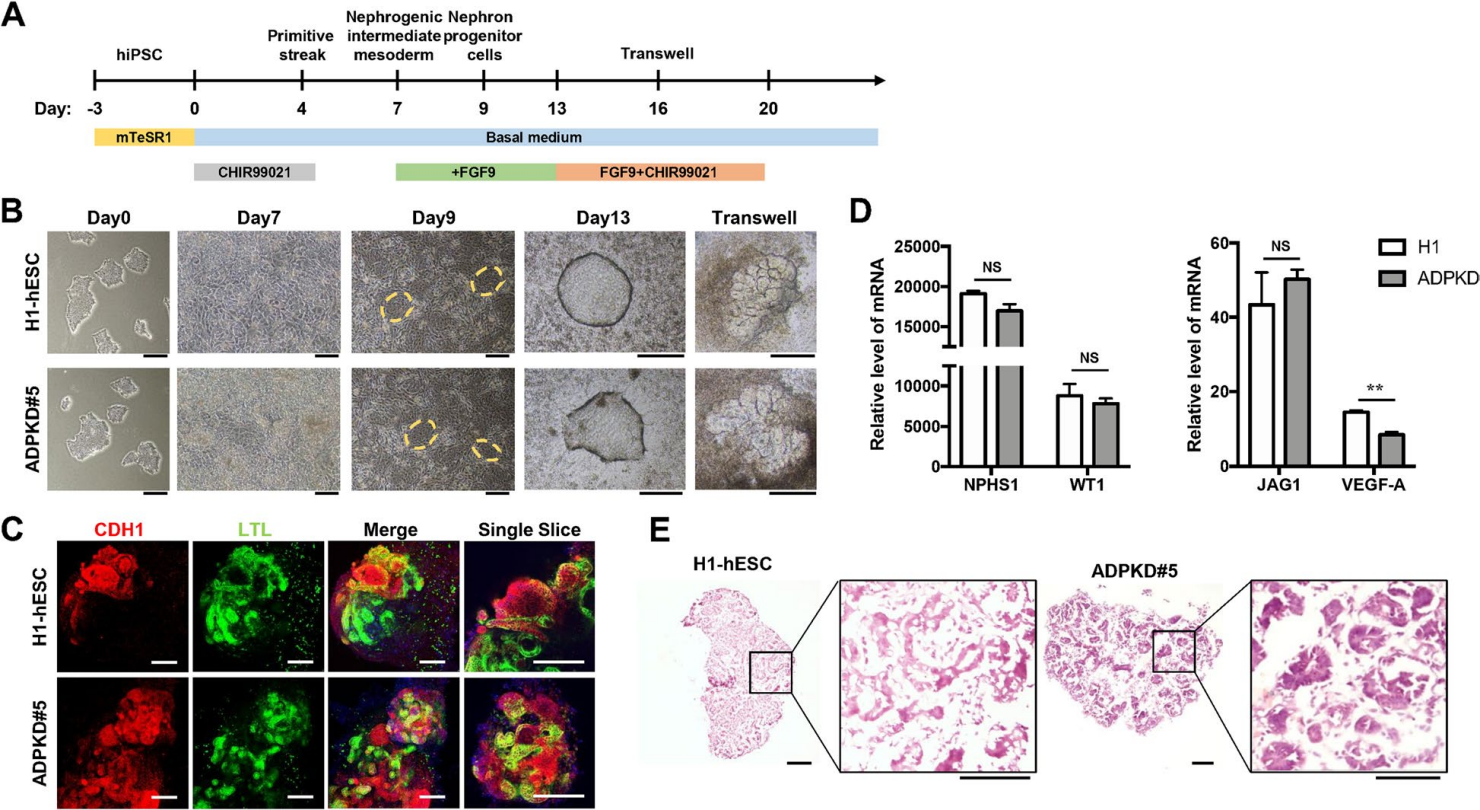
Differentiation and identification of H1 kidney organoids and ADPKD #5 kidney organoids (*PKD1* c.8311 G > A). **A** Workflow of kidney organoid differentiation. **B** Differentiation of H1 hESC and ADPKD hiPSCs into kidney organoids. Representative images at Day 0, Day 7, Day 9, Day 13 and Transwell stage (single mature kidney organoid). Scale bar, 200 μm. **C** Immunofluorescence staining of renal organ-specific marker proteins CDH1 and LTL. Scale bar, 200 μm. **D** Expression levels of the marker proteins NPHS1, WT1, and JAG1 in kidney organoids were identified by RT-qPCR, and the expression levels of vascular marker VEGF-A were also examined. *GAPDH* was used as an internal reference gene. The column represented the average value ± SD (*n* = 3). Statistical significance was determined by the two-tailed Student’s t test. **P < 0.01. **E** HE staining of kidney organoids. The clear tubular structure is visible in the magnified figure. Scale bar, 250 μm

The ADPKD disease phenotype caused by *PKD1* mutations often occurs in the kidney. To investigate whether the *PKD1* mutation (c.8311(G > A)) affects hiPSCs differentiation in vivo, we differentiated ADPKD hiPSCs into kidney organoids to mimic the authentic in vivo conditions of ADPKD (Fig. 3A). Throughout the 20-day differentiation process, we imaged and documented the cellular induction state on Day 0, Day 7, Day 9, Day 13, and Day 20. After 13–15 days of aggregation, 3D kidney organoids were transferred to the upper chamber of the transwell for gas-liquid interface culture until a clear structure appeared in the kidney organoids (Fig. 3B). The expression of renal tubular marker proteins CADHERIN 1 (CDH1) and *Lotus tetragonolobus* lectin (LTL) was then detected by immunofluorescence staining. The confocal image showed that the entire kidney organoid was filled with renal tubular structure, and because CDH1 was labeled in distal renal tubular cells and LTL was labeled in proximal renal tubular cells, the expression signals of the two proteins did not show co-localization (Fig. 3C).In addition, it was found that compared with H1 hESC and ADPKD # 5 hiPSC, the *NPHS1* and *WT1* genes expressed in the glomeruli of each group of organoids were upregulated thousands of times, and *JAG1* and *VEGF-A* were also upregulated tens of times (Fig. 3D). These results further confirmed the successful differentiation of ADPKD hiPSCs into kidney organoids and the presence of angiogenesis in these organoids. In addition, we performed frozen section and HE staining on a single kidney organoid, and the results showed that the tubular structure inside was very dense (Fig. 3E), indicating that the addition of CHIR99021 at the later stage of culture could promote closer and more uniform differentiation of kidney organoids.

### Identification of ADPKD-related disease phenotypes in kidney organoids and renal epithelial cells

**Fig. 4:**
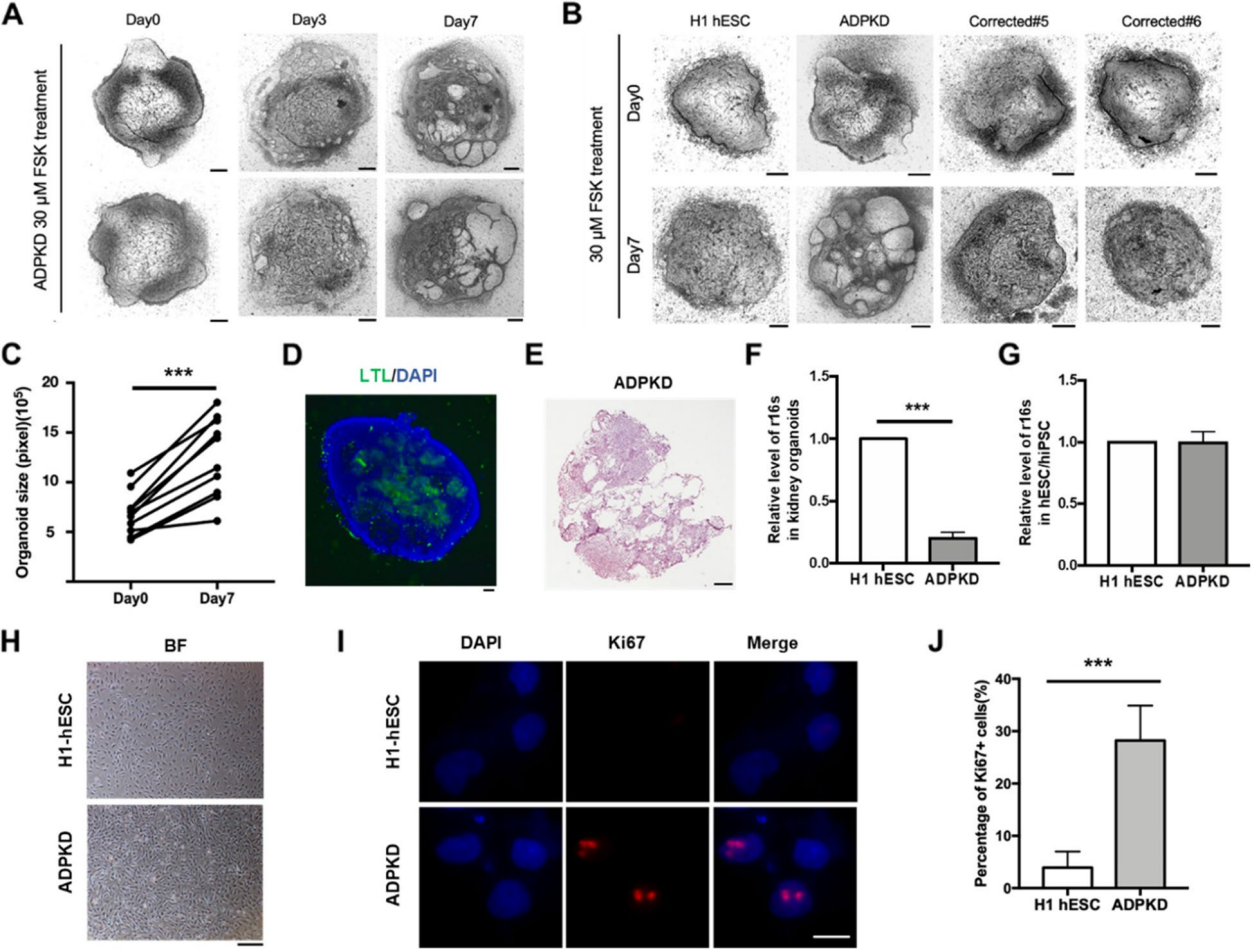
Detection of ADPKD related disease phenotypes in kidney organoids and renal epithelial cells. **A** The bright light image of ADPKD kidney organoids after addition of forskolin for 0, 3 and 7 days. Scale bar, 200 μm. **B** The bright light image of kidney organoids before and after adding forskolin 7 days. Images in each group are the same kidney organoid. The ADPKD group showed obvious cyst formation, while the H1 hESC and corrected grou Immunofluorescence staining p showed no cyst formation. Scale bar, 200 μm. **C** Area statistics of ADPKD kidney organoids after addition of forskolin for 7 days. Error bars indicated SD (*n* = 10). Statistical significance was determined using the two-tailed Student’s t test. ***p < 0.001. **D** Immunofluorescence staining of LTL in the ADPKD kidney organoid. Scale bar, 200 μm. **E** HE staining of ADPKD kidney organoids after addition of forskolin for 7 days. Scale bar, 250 μm. **F** RT-qPCR detected mitochondrial DNA r16S levels in kidney organoids. H1 hESC differentiated kidney organoids as a control. GAPDH as an internal reference. Error bars indicated SD (*n* = 3). Statistical significance was determined by the two-tailed Student’s t test. ***p < 0.001. **G** RT-qPCR detected mitochondrial DNA r16S levels in ADPKD hiPSCs. H1 hESC as a control. GAPDH as an internal reference. **H** Bright-field image of renal epithelial cells. Scale bar, 200 μm. **I** Immunofluorescence staining showed the expression of Ki67 protein in renal epithelial cells. H1 hESC differentiated renal epithelial cells were used as a control. Scale bar, 200 μm. **J** Statistical results of Ki67-positive cells. In each group *n* = 200. Error bars indicate SD. Statistical significance was determined using the two-tailed Student’s t test. ***p < 0.001

Usually, the cAMP signaling pathway is active in the kidney tissue of ADPKD patients. Treating the kidney organoids by adding forskolin to the culture medium could stimulate cAMP signaling, leading to cystic expansion of renal epithelial tissue (Facioli et al. 2021). Therefore, we added 30 μM forskolin to the medium of the kidney organoids. It was observed that conspicuous vesicles emerged in the *PKD1* mutant group after 3 days, and the vesicle phenotype became more pronounced at Day 7 (Fig. 4A). However, this phenotype did not appear in the H1 hESC and ADPKD corrected groups (Fig. 4B). We calculated the area of the kidney organoids and found that the area of ADPKD group generally increased twofold after 7 days of induction (***p < 0.001, Fig. 4C). Immunofluorescence staining revealed that there is no DAPI signal in the area where the vesicles appear and the LTL-labeled renal tubule structure was extruded (Fig. 4D). Frozen sections and HE staining were then performed and the results showed that there were cavities of various sizes inside the ADPKD kidney organoids, and the tubular structure was destroyed (Fig. 4E).

In the kidney tissue from ADPKD patients, *PKD1* mutations lead not only to changes in the biochemical reactions in the mitochondria, but also to a loss in the number of mitochondria in the cells, and the shape of the mitochondria changes from a dense network to a sparse independent individual (Padovano et al. 2018; Ramírez-Sagredo et al. 2021). Therefore, we investigated the number of mitochondria in kidney organoids. qPCR detection of mitochondrial DNA r16S was performed using kidney organoid genomic DNA as a template. Compared to normal kidney organoids, the content of mitochondrial DNA r16S in ADPKD kidney organoids was only 19.98 ± 2.92% (***p < 0.001, Fig. 4F), demonstrating that there was indeed a decrease in mitochondrial copy number. In addition, we also extracted the genomic DNA of H1 hESC and ADPKD hiPSC for qPCR, and found no significant difference between the two groups (Fig. 4G), indicating that the *PKD1* gene mutation has no effect on hiPSCs. The above results prove that we have successfully differentiated kidney organoids with ADPKD disease phenotype using ADPKD hiPSCs.

Accelerated proliferation of renal epithelial cells has been reported in many studies analyzing the cause of vesicle formation in ADPKD disease (Cabrita et al. 2020; Ding et al. 2021). To test whether our differentiated ADPKD kidney organoids have the same phenotype, we transferred the differentiated organoids into 6-well plates for adherent growth, and renal epithelial cells gradually crawled out of the kidney organoids. The remaining kidney organoids were then scraped off and the kidney epithelial cells were digested and passaged for expansion (Fig. 4H). We detected the expression of Ki67 protein in renal epithelial cells by immunofluorescence staining (Fig. 4I). The statistical results showed that Ki67-positive cells accounted for only 3.91 ± 1.55% of normal renal epithelial cells, while in ADPKD renal epithelial cells were as high as 28.22 ± 2.98% (***p < 0.001, Fig. 4J). This illustrated the presence of abnormal proliferation in ADPKD kidney epithelial cells, which is closely associated with vesicle formation.

### Base-editing in renal epithelial cells and kidney organoids using the dual AAV split-ABEmax system

**Fig. 5:**
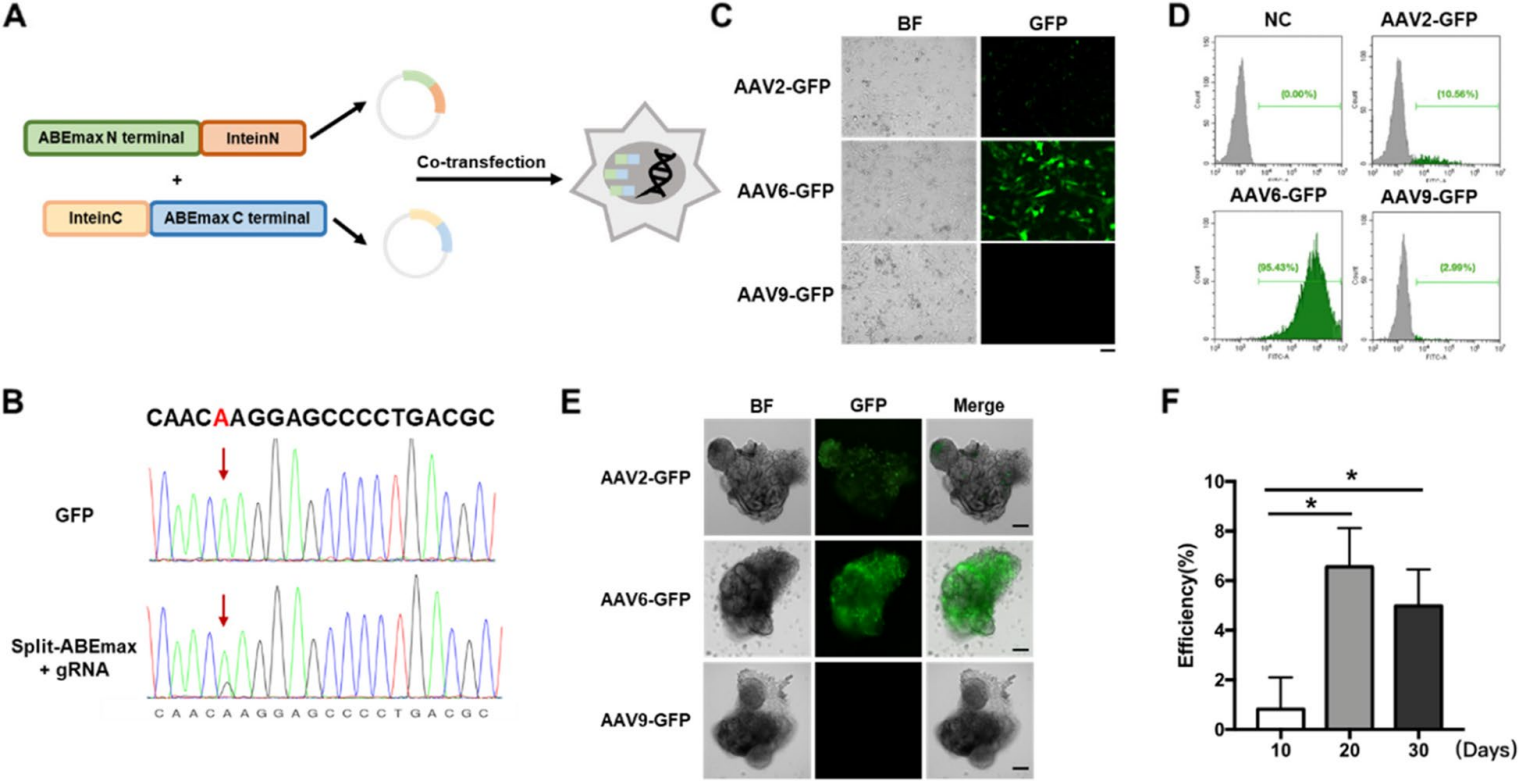
Efficient base editing in kidney organoids using the dual AAV split-ABEmax system. **A** Mechanism of in vivo genome editing by dual AAV delivery of split-ABEmax. **B** Detection of editing efficiency of dual AAV Split-ABE system in HEK293T stable cell line carrying the *PKD1* c.8311(G > A) mutation. Sanger sequencing results of dual AAV Split-ABE system at *PKD1* c.8311 (G > A) in stable cell line. The red arrow marked the c.8311 site. Editing efficiency was calculated using EditR. GFP was used as a negative control. **C** Screening of AAV serotypes in renal epithelial cells. Bright light image and GFP signal of renal epithelial cells after addition of AAV2-GFP, AAV6- GFP, AAV9-GFP for 72 h. Scale bar, 200 μm. **D** Number of GFP-positive cells analyzed by flow cytometry. **E** AAV serotypes screened in the kidney organoids. Bright light image and GFP signal of kidney organoids after addition of AAV2-GFP, AAV6-GFP, AAV9-GFP for 72 h. Scale bar, 200 μm. **F** Editing efficiency in kidney organoids by dual AAV split-ABEmax at 10 days, 20 days, and 30 days. In each group *n* = 10. Error bars indicate SD. *p < 0.05

To edit the *PKD1* (c.8311 G > A) site in cells, a suitable viral delivery vector must first be selected. AAV has the advantages of low immunogenicity, high safety, and long expression time in vivo, which is suitable for gene therapy delivery. As the packaging capacity of AAV was insufficient to load ABEmax, ABE was split into two smaller units by intein-mediated protein trans-splicing (Chen et al. 2020; Wang et al. 2024) (Fig. 5A). Upon co-transfection of dual AAV split-ABEmax vectors into HEK293T-*PKD1* reporter cells, 25% efficient target site correction was observed (Fig. 5B). This result suggests that the split-ABEmax system has effective functionality in the cell.

Different serotypes of AAV have different cell infection efficiencies. There are several serotypes that have been used to infect kidney tissue or cells, such as AAV2, AAV6, and AAV9. To determine which serotype has the highest infection efficiency, three AAV viruses packaged with GFP protein were prepared, namely AAV2-GFP, AAV6-GFP, and AAV9-GFP. Firstly, the three AAV viruses were respectively added to the renal epithelial cells at MOI = 1 × 10^5^ for culture, and the infection efficiency was observed by fluorescence microscopy after 72 h. The cells in the AAV2-GFP group showed sporadic green fluorescence, the fluorescence intensity in the AAV6-GFP group was the strongest, and no fluorescence signal was observed in the AAV9-GFP group (Fig. 5C). We then digested and collected renal epithelial cells infected for 72 h and calculated the proportion of GFP-positive cells by flow cytometry. The results showed that the positive rate of AAV2-GFP group, AAV6-GFP group, and AAV9-GFP group was 10.56%, 95.43%, and 2.99%, respectively (Fig. 5D). The infection efficiency of AAV6-GFP in renal epithelial cells is significantly higher than that of the other two groups.

We also performed the same experiment in kidney organoids, transferring mature single kidney organoids to low adhesion 96-well plates for suspension culture and adding three AAV-GFP viruses at MOI = 1 × 10^5^ separately. The results observed after 72 h were fully consistent with those in renal epithelial cells, with AAV2-GFP showing a weak green fluorescent signal, AAV6-GFP signal present in almost the entire kidney organoids, and AAV9-GFP showing no signal (Fig. 5E). Based on the above results, we selected the AAV6 serotype for subsequent editing experiments. According to literatures, mature kidney organoids can be cultured in vitro for up to 30 days. Considering the complex structure of kidney organoids and the time it takes for ABE to recombine and edit, we detected the editing efficiency at three time points (10 days, 20 days and 30 days). At 10 days, only weak editing was observed in individual samples, with the highest editing efficiency reaching 2.8% (Fig. 5F). At 20 days, the editing efficiencies were significantly improved, with an average of 6.56 ± 0.52% and a maximum of 9.48% (Fig. 5F). The editing efficiencies at 30 days were lower than that at 20 days, with an average of 4.98 ± 0.54% and a maximum of 7.46% (Fig. 5F). In conclusion, our experimental data highlights that the dual AAV-mediated Split ABE system can accurately repair target site mutations in kidney cells and organoids, demonstrating the feasibility of in situ repair of *PKD1* (c.8311 G > A) mutations in patients.

## Discussion

ADPKD is the most common inherited cystic kidney disease, but there is currently no effective cure. This study attempted to use the ABE single-base editor to repair two clinically identified pathogenic *PKD1* point mutations. It was found that only the *PKD1* (c.8311 G > A) site could be efficiently repaired by ABEmax. We generated hiPSCs with the ADPKD-specific mutation (*PKD1* c.8311 G > A) and proceeded to develop disease models by differentiating kidney organoids. ADPKD kidney organoids exhibited clinically similar phenotypes and physiological indicators, and corrected kidney organoids could restore normal phenotypes, providing new data for exploring gene therapy strategies. In addition, we used the dual AAV-mediated Split-ABE system to infect kidney organoids and demonstrated for the first time that ABE can directly repair ADPKD point mutations in kidney tissue, providing a research basis for the clinical treatment of the disease.

Over the past decades, several protocols have been developed to induce iPSCs to differentiate into kidney organoids (Little and Combes 2019; Schutgens et al. 2016; Tekguc et al. 2022). From these, we identified the steps for the differentiation of hiPSCs into kidney organoids and successfully obtained kidney organoids filled with tubular structures that highly expressed DHC1 or LTL. The glomerular marker proteins NPHS1 and WT1 were also highly expressed. In addition, the upper regulation of JAG1 and VEGF-A further confirmed that the presence of angiogenesis in the kidney organoids. Consistent with previous research results (Facioli et al. 2021; Yamaguchi et al. 2000), both undifferentiated iPSCs from ADPKD patient and hESCs control formed similar ectodermal-like spherical structures and did not spontaneously form cysts (Fig. 2). This is because ADPKD is a developmental disease that requires a third independent event (called a third-hit) to accelerate cyst formation and growth of in vivo (Taguchi and Nishinakamura 2017), and it is generally accepted that phenotypes such as an increased vesicle number a size increase with age. The cAMP signaling pathway was activated in ADPKD kidney tissue (Yamaguchi et al. 2000). The addition of forskolin to the culture medium could stimulate cAMP signaling, simulating the in vivo environment of late ADPKD and leading to cystic expansion of renal epithelial tissue (Facioli et al. 2021; Freedman et al. 2015). Indeed, this study found that the *PKD1* mutant group started to appear with obvious vesicles after the addition of forskolin, and as the culture time increased, the vesicle area also expanded, while this phenotype did not appear in the H1 hESC and repair groups (Fig. 4). In addition, the copy number of mitochondria is abnormal in ADPKD kidney organoids. Current administration of the mitochondrial protective peptide (eramipritide) during pregnancy and lactation in mice is not teratogenic and reduces disease severity in both mothers and their affected offspring (Wang and Tran 2022). In addition, the abnormal proliferation of renal epithelial cells has been observed in ADPKD kidney organoids (Fig. 4), which is closely associated with the formation of pathogenic vesicles (Cabrita et al. 2020; Ding et al. 2021).

The development of kidney organoids can serve as a new tool for specific drug screening and disease modeling, especially for rare diseases. Currently, there are studies combining CRISPR/Cas9 genome editing to establish a genetic kidney disease model using isogenic control organoids to complement animal experiments. Researchers have used gene editing technology to rescue the disease phenotype of nephronophthisis (NPHP) (Forbes et al. 2018). Due to genetic and allelic heterogeneity, GC-rich regions and the 5′ region of *PKD1* (exons 1 to 33) being duplicated in six highly homologous pseudogenes, molecular analysis of ADPKD has proven challenging (Carrera et al. 2016; Phakdeekitcharoen et al. 2001; Rossetti et al. 2002). The ADPKD Variant Database (http://pkdb.mayo.edu/) describes > 2000 pathogenic mutations (2110 in *PKD1* and 505 in *PKD2* as of Sep 2, 2023) (Audrézet et al. 2012; Cornec-Le Gall et al. 2013), which including our recent data on an ADPKD-specific mutation (*PKD1* c.8311 G > A). To evaluate the efficacy of gene therapy in ADPKD patients, we performed gene therapy experiments in ADPKD kidney organoids using the dual AAV Split-ABEmax system. The average editing efficiency observed over 20 days was 6.56%, while it was 4.98% over a 30-day period. In our previous study, we found that the editing efficiency of blood vessel organoids was higher at 30 days than at 20 days (Wang et al. 2024). The possible reason for this is that the formation of vascular organoids requires the coating of Matrigel, which presents certain obstacles to the invasion and function of the AAV virus in vascular organoids, whereas kidney organoids are tissues where cells spontaneously aggregate and can directly contact the AAV virus, so that the editing peak is reached at the day 20. On the other hand, it may be that the survival time of kidney organoids in vitro is not as long as that of blood vessel organoids. On day 30, the overall cell activity of the kidney organoids decreased and apoptosis increased.

The use of genome editing tools to treat patients with in vivo genome editing is an important direction for future disease treatment. The delivery method of gene editing tools has a significant impact on the final editing effect. The viral system is an important delivery vector for gene therapy. Compared to retroviruses (RV), lentiviruses (LV), and adenoviruses (AdV), AAV has advantages such as low immunogenicity, high safety, and long in vivo expression time, making it suitable for in vivo gene therapy delivery (Li and Samulski 2020; Wang et al. 2019; Zhi et al. 2022). Regrettably, the low loading capacity of AAV, which is approximately 4.8 kb, limits its utility for essential genome editing tools such as base editors and prime editors (PE) (Anzalone et al. 2020; van Haasteren et al. 2020). The current solution is to split the base editor effector protein into two smaller parts and package them into separate AAVs. Upon infection of the cells, the two separate parts are reconstituted into an active effector protein through RNA trans-splicing or intein-mediated protein trans-splicing mechanisms. Studies have shown that split-ABE reconstructed by intein-mediated protein trans-splicing has more efficient genome editing in vivo (Chen et al. 2020; Levy et al. 2020). Our experiment also proves that this delivery system can perform gene editing normally in kidney organoids. In addition, new ABE variants like ABE8e (Richter et al. 2020), ABE9e (Tu et al. 2022), and ABE9 (Chen et al. 2023) were developed during the trial. Among them, ABE8e shows high editing efficiency. Therefore, it is worth further testing whether they have higher editing efficiency at the *PKD1* mutation sites. Furthermore, the efficiency of gene editing at the delivery level can be improved by screening for more efficient AAV serotypes. For mouse models and subsequent clinical treatments, the efficiency of gene editing can also be improved by multi-point injection to increase treatment efficacy. For other types of point mutations, a new type of DNA base editor that does not rely on any deaminase, the glycosylase-based guanine base editor (gGBE) based on engineered glycosylase, has recently been developed, offering more possibilities for in vivo treatment of genetic diseases such as ADPKD (Tong et al. 2023).

In short, we have successfully established a hiPSC cell line with a previously unreported *PKD1* point mutation. Through induced differentiation, ADPKD kidney organoid and kidney epithelial cell models were obtained and corresponding disease phenotypes were detected, providing a good in vitro model for subsequent exploration of the pathogenesis. Moreover, the dual AAV-mediated split ABE system repaired ADPKD pathogenic point mutations at the organoid level, providing a new alternative treatment strategy for effective cure of single-gene inherited diseases.

## Supplementary Information

Supplementary Material 1.

## Data Availability

Data available on request from the authors.
