# Supplementary material for: Adenine base editor corrected ADPKD point mutations in hiPSCs and kidney organoids
Source: Adv Biotechnol (Singap). 2024 Jun 11;2(2):20. doi: 10.1007/s44307-024-00026-8 (PMC11740842; doi:10.1007/s44307-024-00026-8)
Supplement: Supplementary file 1 — Supplementary Material 1. [file 44307_2024_26_MOESM1_ESM.pdf]

Table S1 Information of PCR and qRT-PCR primers.	
g1198-1-FP	CACCGCTCGGGCCGGCTCCTCGCCC
g1198-1-RP	AAACGGGCGAGGAGCCGGCCCGAGC
g1198-2-FP	CACCGTCGGGCCGGCTCCTCGCCCA
g1198-2-RP	AAACTGGGCGAGGAGCCGGCCCGAC
g8311-FP	CACCGCAACGAGGAGCCCCTGACGC
g8311-RP	CACCGGCGTCAGGGGCTCCTCGTTGC
Loci-PKD1-1198-FP	GTCCTCGGTGCAGAGTGAC
Loci-PKD1-1198-RP	CGATCCACACGTCTAGGCTC
Loci-PKD1-8311-FP	AGGCCTACAACCTGACCTCT
Loci-PKD1-8311-RP	AGGGATTGGAGTCCACCAGA
OT1-FP	AGCCCCTCATTCTGAAGTGC
OT1-RP	GCCTTGTCACTGTGGGCTAA
OT2-FP	GTCGTTCTGGGACTGGAAGG
OT2-RP	CGCACCTCCCAGAAATGACT
OT3-FP	TGGGGAGACTAGTGGGTCAG
OT3-RP	GGGGAGCAGGAGAGATCTCT
OT4-FP	CTGACCCCTTGCCTGAGAAG
OT4-RP	CGGTACTGCTCAGGTGACAG
OT5-FP	TAGGGGATGGAGAAGTGGCA
OT5-RP	AGGAGACCTCATCCACCTGG
OT6-FP	AGGTTGAACTAGTGACGCGG
OT6-RP	CCGTGCTCTCTCTTTGCAGT
OT7-FP	AGGCCTACAACCTGACCTCT
OT7-RP	CACCACGTCACTGAGGTTGG
OT8-FP	AGGCCTACAACCTGACCTCT
OT8-RP	AAAGATGAGCTGCACCACGT
OT9-FP	AAGCCTAGGGGATGGAGAGG
OT9-RP	TCTGCAGGAGACCTCATCCA
OT10-FP	TAGGGGATGGAGAAGTGGCA
OT10-RP	TCTGCAGGAGACCTCATCCA
qP-NPHS1-FP	CACAGCACAGGGTACGAGAG
qP-NPHS1-RP	CAAGAGTCGGGGCTACTCCA
qP-WT1-FP	CACAGCACAGGGTACGAGAG
qP-WT1-RP	CAAGAGTCGGGGCTACTCCA
qP-JAG1-FP	GTCCATGCAGAACGTGAACG
qP-JAG1-RP	GCGGGACTGATACTCCTTGA
qP-VEGF-A-FP	AGGGCAGAATCATCACGAAGT
qP-VEGF-A-RP	AGGGTCTCGATTGGATGGCA
